# Biomarkers and molecular mechanisms of Amyotrophic Lateral Sclerosis

**DOI:** 10.3934/Neuroscience.2022023

**Published:** 2022-11-10

**Authors:** Ashok Chakraborty, Anil Diwan

**Affiliations:** AllExcel, Inc. Shelton, CT, USA

**Keywords:** ALS, *SOD1*, biomarkers, glutamate, protein aggregation, neurodegeneration

## Abstract

Amyotrophic lateral sclerosis (ALS) is a fatal neurodegenerative disease in adults involving non-demyelinating motor disorders. About 90% of ALS cases are sporadic, while 10–12% of cases are due to some genetic reasons. Mutations in superoxide dismutase 1 (*SOD1*), *TAR*, *c9orf72* (chromosome 9 open reading frame 72) and *VAPB* genes are commonly found in ALS patients. Therefore, the mechanism of ALS development involves oxidative stress, endoplasmic reticulum stress, glutamate excitotoxicity and aggregation of proteins, neuro-inflammation and defective RNA function. Cholesterol and LDL/HDL levels are also associated with ALS development. As a result, sterols could be a suitable biomarker for this ailment. The main mechanisms of ALS development are reticulum stress, neuroinflammation and RNA metabolism. The multi-nature development of ALS makes it more challenging to pinpoint a treatment.

## 1. Introduction

ALS, like Parkinson's disease (PD) and Alzheimer's Disease (AD), is known as a non-demyelinating neurodegenerative disease, first described by Dr. Jean-Martin Charcot in 1869 [1] This disease is associated with selective and progressive loss of corticosteroid motor neurons and spinal and bulbar motor neurons. As a result, the symptoms of ALS are muscle cramps, weakness, hyporeflexia and ultimately frontotemporal dementia (DFT), and it eventually leads to death.

A study showed that ALS affects 223,000 people worldwide, and this number may increase by 69% in next 20 years [2]. Therefore, having an understanding and knowledge of early biomarkers and patient follow-ups may improve the prognosis of ALS.

## 2. Etiology

The etiology of ALS remains an enigma, but several genetic, environmental and pathologic clues hold some promise. One finding is that 5% to 10% of patients seem to have inherited ALS in an autosomal dominant pattern. Some of them—2% of the total ALS patients—carry a mutation of a gene on chromosome 21 (Cu, Zn superoxide dismutase [SOD1]) that normally assists in detoxifying superoxide free radicals [3]. ALS is mostly sporadic, however, familial ALS is linked to monogenic causes, such as mutations in *C9orf72, SOD1*, or other genes [4],[5]. Besides, in a study it was shown that tobacco use can also increase the ALS risk by almost four-fold. Other environmental factors such as heavy metals, ambient aromatic hydrocarbons, pesticides and cyanotoxins, as well as head injury, also appear to be a risk factor for ALS [6]–[8]. It therefore appears that genetic as well as environmental factors together or separately may cause the ALS disease [9]–[12].

## 3. Biomarkers

In fact, there are no such reliable biomarkers of ALS, to date [13]. However, mutations in phosphorylated neurofilament heavy chain *(pNfH)* were found to be linked to ALS development [14]. In fact, cerebrospinal fluid (CSF) and blood from victims with ALS and other neurodegenerative diseases showed elevated levels of NFs [14]–[20]. Neurofilament levels actually rise in the blood and CSF ahead of the appearance of disease symptoms in people carrying a mutation in the SOD1 gene [21]. Levels of both neurofilament light chain *(NfL)* and phosphorylated neurofilament heavy chain *(pNfH)* are elevated with poor prognosis in ALS patients [17],[22]–[24]. However, both nonclinical studies with transgenic rodents and clinical studies with familial ALS patients indicate that neuroinflammation and immune dysregulation are related to the pathogenesis and heterogeneity of the ALS disease [6],[25].

Further, activated astrocytes, microglia and monocytes were detected in the motor cortex of ALS patients [26]. Similarly, levels of ferritin, creatine kinase, interleukins, and TNF-α, in plasma of ALS patients were elevated compared to controls, pointing towards the T-cell-affected neuro-muscular pathology in ALS [27]. In addition, C-reactive protein (CRP), an inflammation marker is also elevated in the serum of Pre-ALS and correlates with rapid progression of the disease [28]. Table 1 displays the different biomarkers that are related to different phenotypic abnormalities found in ALS.

**Table 1.** Biomarkers of ALS.

Blood Markers	Related to Inflammation	Related to Metabolic Dysfunction	Related to Neurodegeneration
Elevated in ALS patients: Metalloproteinase-9 (MMP-9) **[29]** Extracellular matrix metalloproteinase inducer (EMMPRIN) **[30]**	Increased circulating levels of: Eosinophil-derived neurotoxin Granzyme A and B High mobility group box 1 (HMGB1) auto-antibody Interleukin-6 Interferon-*γ* Monocyte chemoattractant protein-1 (MCP-1) Tumor necrosis factor-*α* (TNFα) Wide range C-reactive protein (wr-CRP) **[31]–[42]**	Motor neuron pathology Defects in energy homeostasis Weight loss Hypermetabolism, and Hyperlipidemia **[43]**	Loss of motor neurons **[43]**
Increased levels of: MMP-2, another metalloproteinase, correlated with the severity of the disease **[30],[44]**	Decreased levels of: Granulocyte macrophage colony stimulating factor (GM-CSF) OX40 Soluble receptor for advanced glycation end products, and Soluble tumor necrosis factor-related apoptosis-inducing ligand **[45]–[48]**	An increase in: Low- to high-density lipoprotein cholesterol ratio This increased ratio correlated with the survival of ALS patients **[49]**	Increased amounts of: Pro-apoptotic interleukin-1*β* converting enzymes (Caspase-1 and Caspase-9) **[50],[51]**
Low level of: Propeptide of type I procollagen, which is an index of collagen biosynthesis.	Increased concentrations of: Interferon-*γ*, MCP-1, TNF-*α*, and GM-CSF in ALS **[52],[53]** Granzyme B, HMGB1 autoantibody, and wr-CRP **[37],[54],[55]**	APOE concentrations correlated with both the rate of deterioration of the patients and their survival times **[56]**	Increased concentration of cystatin C, which is a cysteine protease inhibitor involved in apoptotic neuronal cell death **[57]**
Increased level of lead in the CSF **[58],[50]**	Decreased expression of C-C chemokine receptor type 2 (CCR2) in monocytes of ALS patients **[34],[60]**	Increased levels in ALS: The circulating concentration of N-acetylaspartate **[61]**	High levels of neurofilament light chain in the serum and CSF of ALS patients **[62],[63]**
Mutations in TAR DNA-binding protein 43 *(TDP-43)* cause an accumulation of *TDP-43* in the cytoplasm of circulating lymphomonocytes from ALS patients **[64]–[67]**	Decreased expression of human leukocyte antigen by ALS monocytes **[60]**		Increased expression of phosphorylated neurofilament heavy chain *(pNfH)* in ALS patients **[68],[69]**
Binding of mutant *C9orf72* to trimethylated histones was detected in ALS mononuclear cells **[70]**	Increased amount of: Natural killer T lymphocytes **[69]** Neutrophil-to-lymphocyte ratio **[55]** and, Decrease in the number of regulatory T cells **[60],[71]**		Increased expression of phosphorylated neurofilament heavy chain *(pNfH)* in CSF of ALS patients **[72],[73]**

## 4. Genetic factors in ALS

More than 20 genes have been described for familial ALS (fALS) cases. However, those gene products are very different in their functions and make it difficult to find a clue for the onset of ALS disease. In most cases, the cause of sALS is not known, but it generally starts at an older age [9]–[11]. Several fALS genes such as *SOD, TDP-43, FUS* and *C9ORF72* have also been reported in sALS cases [74].

**Other Rare Occurring Mutant Genes in fALS:**

- A missense mutation in the D-amino acid oxidase (*DAO*) gene has been reported in several families with ALS disease [75]. *DAO* mutations decrease the cell viability, increase the ubiquitinated aggregates and enhance the apoptosis of primary motor neurons in culture [75],[76].
- In one case, a genetic subtype *ALS7* is found to be linked to chromosome 20ptel-p13 and shows the signs of onset of fALS [10].

Tables 2 and 3 display the responsible genes involved for fALS and sALS disease, respectively.

**Table 2.** Responsible Gene Factors for the Onset of fALS Disease.

Genetic Factors	fALS
*SOD1* (Superoxide dismutase 1)	Mutation of the *SOD1* gene found in ALS interrupts the cellular detoxification and results in free radical toxicity and cell death **[77]**. Mutations in SOD1 have been reported in ~20% of fALS and in ~1-4% of sALS **[10],[78]**.
*TARDBP* (TAR DNA binding protein)	*TDP-43* gene product binds to DNA and RNA and thus participates in the transcription and splicing of RNA. Mutation of *TDP-43* was found in ALS cases **[79]–[81].**
*FUS* (Fused in sarcoma)	*FUS* is a DNA- and RNA-binding protein, and it is involved in mRNA transport to neuronal dendrites. Mutation of this gene are found in ALS **[82]–[85].**
*C9ORF72* (Chromosome 9 open reading frame 72)	The repeat expansions of the *c9orf72* gene are found in the pathogenesis of ALS **[86].**
*VAPB* (Vesicle-associated membrane protein-associated protein B)	An aggregated loss-of-function mutant of *VAPB* predisposes motor neurons to ER stress-related death in ALS **[87].**
*NEK1*	Discovered in 2016, mutations in *NEK1* are present in both sporadic and familial forms of ALS. Together, *NEK1* is associated with 3% of all ALS cases **[88].**
*UBQLN2*	Ubiquilin-2 (*UBQLN2*) resides on the X chromosome. Mutations in the gene interfere may lead to the accumulation of harmful material within the cell. Both men and women may develop ALS due to ubiquilin-2 mutations **[89]**
*KIF5A*	*KIF5A*, or kinesin family member 5A, involved in transport of protein cargo in the cell. Mutations contributing to familial ALS appear to be inherited in an autosomal dominant fashion **[88].**
*VCP* (valosin-containing protein)	VCP is a hexameric type II ATPase of the AAA family involved in multiple cellular functions. Immunohistochemical study of VCP in the skin from patients with ALS and controls reveals that the proportion of *VCP*-positive cells in the epidermis in ALS is higher than that in controls **[90].**
*ALS2* (alsin)	It promotes neurite outgrowth in cell cultures through activation of the small GTPase **Rac1 [91].** Alsin knock-out mice showed increased vulnerability to oxidative stress, that causes motor neuron degeneration **[92],[93].**
*SETX* (senataxin)	SETX mutations-caused motor neuron degeneration may result from the aberrant RNA processing **[94].**
*ANG* (angiogenin)	ANG mediates neovascularization and promotes neurite outgrowth during early embryonic development. Mutations in ANG gene cause an onset of the classic signs of ALS **[95].**
*OPTN* (optineurin)	OPTN is co-localized with FUS, TDP43 and SOD1 in inclusion bodies of sALS and fALS **[96].**
*SPG* (spatacsin)	*SPG* is the most common form of recessive fALS with juvenile onset **[97],[98].** The accumulation of spatacsin in non-myelinated axons suggesting axonal transport disturbance **[99].**
*FIG 4* [phosphoinositide 5-phosphatase that regulates PI(3,5)P2]	*FIG 4* is a signaling lipid that helps in retrograde trafficking of endosomal vesicles to the trans-Golgi network **[100].** Mutations in *FIG 4* result in neurodegeneration in sensory and autonomic ganglia, motor cortex and striatum **[100]–[102].**
*SIGMAR1* (SIGMA Non Opiod Intracellular Receptor1)	The *SIGMAR1* protein functions as a subunit of the ligand regulated potassium channel, which can bind to neurosteroids, psychostimulants, and dextrobenzomorphans **[103].** A mutation in *SIGMAR1* gene established a connection between familial ALS with FTD to chromosome 9p13.2-21.3 **[104],[105].**
*DCTN1* (Dynactin)	Mutations have been identified in *DCTN1* gene in sALS, fALS and ALS-FTD families **[106].** *DCTN1* mutations cause neurodegeneration by impairing axonal transport in motor neurons **[107],[108].**

**Table 3.** Responsible Gene Factors for the Onset of sALS Disease.

Genetic Factors	sALS
*APEX1* (Apurinic endonuclease)	*APEX1* participates in the process of DNA repair and DNA binding of transcription factors and plays a protective role against oxidative stress, and the mutants lose redox activity and fail to stimulate cell proliferation [109]
*CHMP2B* (Charged multivesicular body protein 2B)	*CHMP2B* mutations lead to dendritic retraction and autophagosomal aggregation in cortical neurons and in hippocampal neurons, implying that *CHMP2B* is needed for dendritic spine growth and maturation [110], [111].
*NEFL* (Neurofilaments)	*NEFL* is required for neurofilament assembly. Mutations in it are known to cause a form of hereditary, sensory and motor neuropathy [112]. Homozygosity for the short repeat allele is associated with sALS [113]. Deletions and insertions in the C-terminal KSP repeats of *NEFL* are noted in some sALS patients [114].
*PON* (paraoxonases)	*PON* proteins are involved in the detoxification of organophosphate pesticides, neurotoxins and aromatic esters. Mutation in this gene causes neurotoxicity [115]. Seven mutations in the *PON* genes have been found in patients with fALS and sALS [116].
*PRPH* (Peripherin)	*PRPH* acts as a cytoskeletal protein, is present in the neurons of the peripheral nervous system and helps in axonal regeneration [117]. Overexpression of wild-type *PRPH* in transgenic mice develops a selective, large scale late-onset motor neuron degeneration characterized by intermediate filament inclusions [118]. Two homozygous missense mutations have been identified in *PRPH* gene which may contribute to the ALS pathogenesis [119].
*NEK1*	Discovered in 2016, mutations in *NEK1* are present in both sporadic and familial forms of ALS. Together, *NEK1* is associated with 3% of all ALS cases [88].
*ATXN2* (Ataxin-2)	*ATXN2* form a RNA-dependent complex with *TDP-43* and leads to enhanced dislocation of *TDP-43* into the cytoplasm in the spinal cord motor neurons in ALS patients [120].
*PGRN* (Progranulin)	It is a glycoprotein, linked to tumorigenesis and activated microglia in several neurodegenerative diseases [121]. To date, only a single study links *PGRN* mutations to ALS [122].
*VEGF*	*VEGF* can cause the late-onset motor neuron degeneration similar to ALS [123]. Spinal cords of ALS patients show reduced expression of VEGF and its receptor [124]. Certain SNPs in the VEGF gene are associated with the lower level of VEGF expression and higher risk of ALS, suggesting a link between VEGF levels and ALS susceptibility [125].
*SMN-1, SMN-2* (Survival motor neuron)	*SMN* has an important function in mRNA metabolism. The impaired assembly and function of the spliceosome formed by *SMN* and associated protein could cause motor neuron degeneration [126],[127] Homozygous deletion mutations in *SMN* genes are not found in ALS but an abnormal copy numbers in *SMN1* could increase the risk for ALS [128].

## 5. Molecular mechanisms of amyotrophic lateral sclerosis

**Figure 1.**
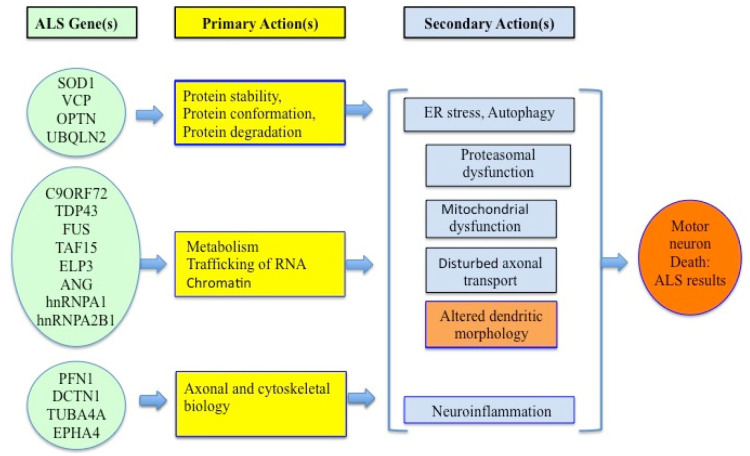
A Schematic Diagram of ALS Pathology.

The common ALS genes, listed in Fig. 1, define three primary actions in ALS pathophysiology:

- **Protein conformational instability and its degradation:** Loss of antioxidant defense (*SOD 1* function) causes the accumulation of free radicals and generates oxidative stress [78],[88]. Aggregation of proteins, *SOD1* [present only in the fALS] [77],[129], *TDP-43* [130], *FUS* [131],[132], Optineurin (*Optn*), Ataxin-2 and Ubiquilin-2 [129] are involved in causing ALS.
- **Impaired trafficking of RNA:** Mutation of multiple ALS genes showed disturbances in RNA-binding proteins, RNA synthesis, its function and metabolism. Mutations in the *TDP-43, FUS and C9orf72* genes develop stress granules in the cytoplasm, toxicity to neurons and disturbance of the splicing activity [133].
- **Altered axonal and cytoskeletal biology:** Cytoskeletal dynamics are altered in ALS. Mutations in profilin-1 are likely to impair energy-dependent extension of filamentous actin and elongation of growth cones, a process that is enhanced by a reduction in signaling from ephrin A4. Tubulin mutations compromise the structure of microtubules. Mutations in dynactin are predicted to impair retrograde transport along the microtubule backbone.

All those above disturbances culminate to multiple secondary, downstream pathologic processes, including activation of endoplasmic reticulum (ER) stress and autophagy, proteasomal as well as mitochondrial dysfunction, disturbed axonal transport, altered dendritic morphology and excitotoxicity.

- **Reticulum stress:** It is induced by the accumulation of abnormal proteins due to mutations of *SOD1* in ALS [134],[135].
- **Structure and Functioning of Mitochondria:** Alterations in the vacuolization and mitochondrial swelling decreases in the activity of the respiratory chain and causes ALS [136].
- **Glutamate Excitotoxicity:** Glutamate is a powerful neurotransmitter, synthesized at the presynaptic terminal and is diffused to activate post-synaptic neuron AMPA (α-amino-3-hydroxy-5-methyl-4-isoxazolepropionic acid) and NMDA (N-methyl-D-aspartate). In ALS patients, glutamate levels were abnormally high in their plasma compared to healthy subjects. This phenomenon may cause neuronal toxicity and cell death in ALS [137]–[139].
- **Neuroinflammation:** As the disease progresses, microglial cells acquire an M1 phenotype and secrete ROS, pro-inflammatory cytokines and neurotoxic molecules, and ultimately promote motor neuron death [140],[141].

Therefore the proposed pathogenic mechanisms may include either protein aggregation, oxidative stress, mitochondrial dysfunction, glutamate receptor-mediated excitotoxicity or neuroinflammation [2],[4],[5],[9],[10]. In Fig. 1, we have shown by a schematic diagram how and where the genes are involved in ALS pathology.

*Conformational instability and aggregation of proteins, impaired trafficking of RNA and altered axonal and cytoskeletal dynamics are the primary ones of all the responsible genes mutations These result on multiple secondary, downstream pathologic processes such as activation of endoplasmic reticulum (ER) stress and autophagy, proteasomal* excitotoxicity*, altered mitochondrial function, disturbed axonal transport, altered dendritic morphology, and neuroinflammation*.

## 6. Management oof ALS cases

The diverse pathophysiology in ALS limits the treatment strategies for the management of the disease and therefore demands the cohort treatment through neurologists, pneumologists, physiotherapists, nutritionists, etc. FDA (U.S. Food and Drug Administration) has approved, so far, only two drugs to be applied for the treatment of ALS patients. One is Riluzole, one of whose action is to inhibit the release of glutamic acid from neurons, *in vivo*, and thus blocks the postsynaptic effects [142]. It may be partly due to inactivation of voltage-dependent sodium channels on glutamatergic nerve terminals, as well as activation of a G-protein-dependent signal transduction process or noncompetitive blockade of N-methyl-D-aspartate (NMDA) receptors [142]. *In vivo*, riluzole actually showed neuroprotective, anticonvulsant, and sedative properties. It improves survival by a couple of months, only [143]–[148], whereas another drug, Edaravone, which is a free radical scavenger, reduces oxidative stress and inhibits neuronal death in animal models [149]. In clinical trial, this drug, Edaravone, showed promising results in decreasing the death rate ofan ALS ALS patient by 35–40% and leading to approval in the United States in 2017 [150].

Very recently (Sept. 2022) the U.S. Food and Drug Administration approved Relyvrio (sodium phenylbutyrate/taurursodiol) to treat patients with fatal ALS disease despite of uncertainty about its effectiveness *(https://www.cnn.com/2022/09/29/health/als-drug-relyvrio)*. Relyvrio targets both endoplasmic reticulum (ER) and mitochondria of motor neurons in ALS patients. Vitamin E (tocopherols and tocotrienols) as an antioxidant can slow down the onset, and also the progression of ALS disease [151].

## 7. Discussion

ALS is a neurodegenerative disease that starts due to defective function or non-function of motor neurons in the spinal cord and in the brain. Symptomatically the disease is characterized by progressive muscular atrophy, slow speech, paralysis, swallowing disturbances and respiration problems [152]. In most cases, death occurs typically 3–5 years after the diagnosis of the disease as the failure of the respiratory system becomes prominent, although in some cases survival could be longer [153]. From a genetic point of view, the majority of ALS cases are sporadic (sALS), and approximately 10% of cases can be considered familial (fALS). ALS is a complex disorder, and the biological mechanisms are still not completely understood as it involves different pathways including abnormal RNA metabolism, altered mitochondrial function and regulation of oxidative balance, modulation of neuronal excitability, axonal transport, control of the inflammatory response and protein folding and degradation, in the disease pathogenesis [154],[155].

In ALS, as in other neurodegenerative diseases, there is an urgent need for sensitive, reliable diagnostic and disease-progression biomarkers for early detection and treatment of the disease. Peripheral blood inflammatory cytokines as they are increased in other neuro-degenerative disease, cannot be considered as a specific diagnostic marker for ALS.

Many anti-inflammatory molecules have been used against ALS over the past 3 years with some success, but a cure is still far away. The limitations of sample collections for diagnostic marker studies are as follows:

- (1) Collection of disease samples and controls should be with the same demographic characteristics
- (2) The collection of samples at different days rather than at a single time point on any single day should be better as biomarkers of disease progression.
- (3) The sensitivity of the used technique, other than ELISA, should be considered to detect the minimal concentrations of the molecule suspected for the disease.

Plasma cytokines are elevated in ALS patients and are still considered as a disease marker for progression and for disease severity [156], however, more knowledge are needed to investigate a possible role of some other inflammatory cytokines those could be used for diagnosis of the disease as well its prognosis. However, blood biomarkers might not reflect the motor neuron defects as those present in the CSF [157]. In fact, the blood-brain barrier could inhibit the crossing of disease biomarkers towards the systemic compartment. Since frequent collection of CSF is hazardous we have to rely on blood samples as an ideal source of biomarkers.

## 8. Conclusion

The genetic spectrum of fALS and sALS is heterogeneous. Several genes in ALS are known to cause many other neurodegenerative diseases, such as *alsin* with primary lateral sclerosis (PLS), and infantile onset ascending hereditary spastic paralysis (IAHSP), senataxin with SCAR1 or AOA2, spatacsin with HSP, VAPB with SMA, FIG 4 with CMT type 4 J, OPTN with primary open angle glaucoma. In addition, ALS and FTLD are similar to each other from their clinical as well as pathological points of view. A number of autosomal-dominant genes have also been described for ALS or FTD such as VCP, and TARDBP. The presence of two neurodegenerative phenotypes within the same family and even within the same individual naturally raises questions about the genetic and environmental interaction on the disease initiation.

Using linkage analysis, candidate gene studies and genome wide association studies, about 1/3 fALS and a small number of sALS have been revealed the disease-caused genes. However, despite all those analyses, the cause of major sALS cases remains unknown. Emphasis should be given on gene-environment interactions and crosslink in ALS, as the majority (90%) of the cases are sporadic in origin. The identification of novel genes and their modifiers may advance this research and may enable us to find a new treatment for ALS, in the near future.
